# Bcl-2 expression and prognostic significance in feline invasive mammary carcinomas: a retrospective observational study

**DOI:** 10.1186/s12917-018-1772-x

**Published:** 2019-01-10

**Authors:** Elie Dagher, Jérôme Abadie, Delphine Loussouarn, Dominique Fanuel, Mario Campone, Frédérique Nguyen

**Affiliations:** 1 AMaROC, Oniris (Nantes Atlantic College of Veterinary Medicine, Food Science and Engineering), Oniris site Chantrerie, CS40706, 44307, Cedex 3 Nantes, France; 2 CRCINA, INSERM, Université d’Angers, Université de Nantes, Nantes, France; 3 Hôtel-Dieu CHU de Nantes, Anatomie Pathologique, cedex 01, Nantes, 44093 France; 4 Integrated Center for Oncology, ICO, 15 rue André Boquet, cedex 02, 49055 Angers, France

**Keywords:** Feline mammary carcinoma, Bcl-2, Spontaneous animal model, Breast cancer

## Abstract

**Background:**

Cats spontaneously develop invasive mammary carcinomas with high clinical aggressiveness, and are considered relevant animal models for human breast cancer. Bcl-2 is an anti-apoptotic pro-survival protein, whose expression is associated with a favorable outcome in human breast cancer. The aim of our study was to determine the frequency of Bcl-2 expression in feline invasive mammary carcinomas (FMCs), its relationship with other clinicopathologic variables, and its prognostic value. This retrospective study included 180 FMCs, diagnosed in female cats treated by surgery only, with a 2-year follow-up post-mastectomy. Bcl-2, ER, PR, Ki-67, HER2, and CK5/6 expression were determined by automated immunohistochemistry. A receiver-operating-characteristic curve was used to set the threshold for Bcl-2 positivity.

**Results:**

The cohort comprises 32% (57/180) luminal FMCs defined by ER and/or PR positivity, and 68% (123/180) triple-negative FMCs (negative for ER, PR, and HER2). Bcl-2 expression was considered as positive when at least 65% of tumor cells were immunohistochemically stained. Thirty-one out of 180 FMCs (17%) were Bcl-2-positive. There was no significant association between Bcl-2 expression, and the tumor size, nodal stage, histological grade, or ER, PR, Ki-67, HER2, and CK5/6 expression. By multivariate survival analysis (Cox proportional-hazards regression), Bcl-2 positivity in FMCs was associated with longer disease-free interval (*p* = 0.005, HR = 0.38), overall survival (*p* = 0.028, HR = 0.61), and cancer-specific survival (*p* = 0.019, HR = 0.54) independently of other powerful prognostic factors such as pathologic tumor size, pathologic nodal stage, and distant metastasis. The positive prognostic value of Bcl-2 was confirmed in both luminal FMCs, of which 9/57 (16%) were Bcl-2-positive, and in basal-like triple-negative (ER–, PR–, HER2–, CK5/6+) FMCs, of which 14/76 (18%) were Bcl-2-positive.

**Conclusions:**

Compared to human breast cancer, Bcl-2 positivity in feline invasive mammary carcinomas is also associated with better outcome, but is less common, and not associated with ER, PR, and HER2 expression. Cats with spontaneous Bcl-2-positive FMCs could be useful in preclinical trials evaluating anti-Bcl-2 strategies for chemoresistant luminal or triple-negative breast cancers.

## Background

The B cell lymphoma/leukemia-2 (*BCL2*) gene, first discovered in B-cell malignancies [1], encodes the integral outer mitochondrial membrane protein Bcl-2 that is expressed by a wide range of normal tissues and tumors, and regulates the intrinsic mitochondrial pathway of apoptosis [2–4].

As an anti-apoptotic pro-survival protein, Bcl-2 overexpression in human and feline cancers would be expected to be associated with a more aggressive behavior. However in human breast cancer, it was proven in different studies that Bcl-2 expression is associated with a favorable outcome [2, 5] in terms of disease-free interval [6–10], overall survival [6, 8, 10–13] and specific survival [7, 14]. Hence Bcl-2 is reported to exert two opposing activities, one (anti-apoptotic) that promotes tumorigenesis, and another (anti-proliferative through G0 arrest), which is anti-tumorigenic [15]. Many studies in human breast cancer have explored the protective effects of Bcl-2 positivity in luminal breast cancers, i.e., which are positive to Estrogen Receptor alpha (ER) and/or Progesterone Receptor (PR), and have found that Bcl-2 positivity is associated with a better outcome [7, 16–19]. However, few studies have explored the prognostic significance of Bcl-2 in triple-negative breast cancers, i.e., those that are negative to ER, PR, and Human Epidermal growth factor Receptor-2 (HER2). Some studies have reported that Bcl-2 positivity of triple-negative breast cancer is associated with better cancer-specific survival and disease-free survival [20], while other studies showed that Bcl-2 positivity is associated with a worse prognosis in terms of overall survival [13–21], and finally some authors did not find any prognostic significance of Bcl-2 in triple-negative breast cancer [12].

Mammary tumors are common in cats and constitute approximately 17% of all feline neoplasms [22, 23]. Among feline mammary tumors, reported malignancy rates range between 80 and 90% [24]. Feline mammary carcinomas (FMCs) have a tendency to be biologically aggressive, with a median reported overall survival time of 8–12 months post-diagnosis in most studies with follow-up [25–30]. Bcl-2 expression has been described in feline mammary carcinomas, in a study where 13 out of 15 carcinomas were Bcl-2 positive, however without associated survival analysis [31]. To the best of our knowledge, this is the first study that investigates the prognostic significance of Bcl-2 in feline mammary carcinomas.

The aims of this study were 1) to determine the frequency of Bcl-2 expression in feline invasive mammary carcinomas, 2) to correlate Bcl-2 expression with other clinicopathologic characteristics, and 3) to investigate the prognostic significance of Bcl-2 expression in triple-negative and luminal feline invasive mammary carcinomas.

## Methods

### Animals, inclusion criteria, and follow-up study

This retrospective study included 180 cats with invasive mammary carcinomas, diagnosed between 2007 and 2010 at two diagnostic facilities in veterinary pathology: the “Laboratoire d’Histopathologie Animale” (LHA) at Oniris, Nantes, and the “Laboratoire d’Anatomie Pathologique Vétérinaire” (LAPV) of Amboise, France. The owners’ written consent and approval from the local animal welfare committee were obtained prior to inclusion.

Criteria for inclusion were (1) a diagnosis of invasive mammary carcinoma, confirmed by an absent layer of p63-positive myoepithelial cells by immunohistochemistry (monoclonal mouse anti-p63 antibody, clone 4A4, abcam ab111449) that differentiates invasive from in situ breast ductal carcinoma [32, 33], (2) in a female cat; (3) absence of other neoplasm evident at the time of diagnosis, (4) the animal was treated solely by surgery (no chemotherapy or radiation therapy pre or post mastectomy), and (5) follow-up was available for at least 48 months post-surgery.

The collected clinical information included age at diagnosis, breed, spaying status, history of contraception and parity, medical history, the location of the mammary carcinoma (mammary glands M1 to M4, side, multicentricity), and evidence of distant metastases determined by medical imaging (radiography and/or ultrasonography), classified into three categories: MX (no medical imaging performed), M0 (medical imaging revealed no distant metastasis), and M1 (presence of distant metastases).

All 180 cats were followed up for at least 2 years (for those animals still alive), to determine the disease-free interval (DFI, interval from mastectomy to the earliest local recurrence, new primary tumor, lymph node metastasis and/or distant metastasis), overall survival (OS, time between mastectomy and death from any cause), and cancer-specific survival (SS, time between mastectomy and death attributable to the mammary carcinoma).

For disease-free interval analysis, censored cases corresponded to animals in which no locoregional recurrences and no distant metastases were reported during the follow-up period. For overall survival analysis, censored cases corresponded to animals that were alive at the end of the follow-up period, which in this study was not shorter than 730 days (2 years). For specific survival analysis, censored cases corresponded to cats that were alive at the end of the follow-up, or died from an unknown cause, or died from a cause that was not related to their mammary carcinoma.

### Histologic methods and criteria

Each mammary tumor was fixed in 10% neutral buffered formalin, embedded in paraffin, and cut into 3 μm-thick sections for Hematoxylin-Eosin-Saffron (HES) staining. In case of multiple (within a given mammary gland) or multicentric (within different mammary glands) invasive mammary carcinomas, the carcinoma with the largest diameter on histological section was selected for analysis. The recorded histological types included tubular, papillary, tubulopapillary, solid, adenosquamous, anaplastic, cribriform, mucinous mammary carcinomas and comedocarcinomas, with most of the cases demonstrating more than one growth pattern. In these instances, the largest growth pattern of the tumor determined the subtype.

The pathologic tumor size (pT) was measured on HES-stained histological sections as the largest tumor dimension, in millimeters. Lymphovascular invasion (LVI), the presence of lymph and/or blood vessel emboli, was assessed on the HES-stained sections. Central necrosis and squamous differentiation were assessed as present/absent regardless of extension. Tumor-associated lymphohistiocytic inflammation was quantified as absent (0), minimal (1), mild (2), moderate (3), marked (4), or severe (5), and then considered negative for scores 0–2 and positive for scores 3–5. To be considered moderate, peritumoral mononuclear inflammation had to involve at least half of the circumference of the mammary carcinoma; marked peritumoral inflammation had to be multifocally observed along all the circumference; severe peritumoral inflammation had to contain nodular lymphohistiocytic infiltrates resembling lymphoid follicles.

An inguinal or axillary lymph node was removed from 126 cats (70%) during mastectomy, and the presence or absence of nodal metastases, regardless of their size, was assessed on standard HES histological sections. An anti-pancytokeratin immunohistochemistry (monoclonal mouse anti-human cytokeratins, clones AE1-AE3, Dako M3515) was performed in all cases that were initially pN0 (negative pathologic nodal stage) on HES histological sections. This led to reclassify some initially pN0 cases to pN+ (positive pathologic nodal stage) because they included isolated tumor cells (< 0.2 mm in diameter) or micro-metastases (0.2–2.0 mm in diameter). The pathological nodal stage also included a pNX category (lymph node not sampled for histopathology).

FMCs were graded according to the Elston and Ellis histological grading system for human breast cancer [34], relying upon 3 criteria, each scored 1–3 points: the percentage of tubule formation, quantified at low-power magnification as a percentage of the tumor parenchyma; the degree of nuclear pleomorphism, assessed at high-power (400x) magnification in the least differentiated and/or most invasive portion of the tumor, typically along the periphery; and the mitotic count in 10 high-power fields (400x, field diameter 0.625 mm). Grade I (3–5 points), II (6 or 7 points), and III (8 or 9 points) carcinomas corresponded respectively to well-differentiated, moderately differentiated, or poorly differentiated carcinomas.

Tumors were also graded according to the mitotic-modified Elston and Ellis (MMEE) grading system proposed by Mills et al. [35], in which the mitotic count categories of the EE grading system were modified to better accommodate the wide range and high magnitude of mitotic counts observed within feline mammary carcinomas. Mitotic figures were counted in 10 consecutive fields at the periphery of the tumor in the areas of highest proliferative activity. According to Mills’ publication [35], the thresholds for the mitotic counts were ≥ 51 and ≥ 71 mitoses in ten high-power fields.

FMCs were staged according to the modified WHO staging system [36, 37] into: stage I, i.e., primary tumor less than 2 cm in diameter (pT1) with no evidence of regional or distant metastases (pN0-pNX and M0-MX); stage II, i.e., primary tumor 2 to 3 cm in diameter (pT2) with no evidence of regional or distant metastases (pN0-pNX and M0-MX); stage III, i.e., pT1-pT2 with evidence of regional metastases (pN+), or primary tumor greater than 3 cm in diameter (pT3), any pN but without evidence of distant metastases (M0-MX); and stage IV, i.e., evidence of distant metastases (M1) regardless of tumor size or nodal stage.

### Immunohistochemistry

Automated immunohistochemistry (Benchmark XT, Ventana Medical Systems, Roche Diagnostics) was used to detect Estrogen Receptor alpha (ERα), Progesterone Receptor (PR), Human Epidermal Growth Factor Receptor 2 (HER2), the proliferation marker Ki-67, Cytokeratins 5 and 6 (CK5/6), and Bcl-2 (Table 1). The detection systems were the iView DAB detection kit (Ventana Medical Systems, Roche Diagnostics, 760–091) for ERα, PR, Ki-67 and CK5/6, chosen to minimize background staining; the UltraView Universal DAB detection kit (Ventana Medical Systems, Roche Diagnostics, 760–500) for HER2, as recommended by the manufacturer; and for Bcl-2, the OptiView DAB IHC Detection Kit (Ventana Medical Systems, Roche Diagnostics, 760–700), which is a 3-step indirect, biotin-free system, chosen for its high sensitivity, and absence of background staining in feline tissues. The three detection systems use horseradish peroxidase with hydrogen peroxide substrate, and 3,3′-diaminobenzidine tetrahydrochloride (DAB) chromogen.

**Table 1:** Summary of antibodies and conditions of use

Antigen	Clone and origin	Dilution, incubation time	Source, reference	Antigen retrieval
ERα	C311 mouse monoclonal	1:50 44 minutes	Santa Cruz Biotechnology, sc-787	None
PR	10A9 mouse monoclonal	1:50 1 h40	Meridian Life Science, K42546 M	HIER, CC1, 1 hour
HER2	4B5 rabbit monoclonal	Prediluted 8 minutes	Roche Diagnostics, 790–2991	HIER, CC1, 30 minutes
Ki-67	MIB1 mouse monoclonal	1:50 32 minutes	Dako, M7240	HIER, CC1, 1 hour
Cytokeratins 5/6	D5/16 B4 mouse monoclonal	1:50 16 minutes	Dako, M7237	HIER, CC1, 30 minutes
Bcl-2	7/Bcl-2 mouse monoclonal	1:50 44 minutes	BD Transduction laboratories, 610,539	HIER, CC1, 24 minutes

*CC1* cell conditioning solution 1, Ventana Medical Systems (reference 950–124)

*HIER* heat-induced epitope retrieval

Negative controls for IHC were included in each run, and consisted in replacing the primary antibody with normal rabbit or mouse serum (prediluted reagents, Roche Diagnostics). The positive controls were internal controls in most cases (i.e., skin epidermis and hair follicles for Ki-67; mammary gland surrounding the carcinoma for ERα, PR, CK5/6 and Bcl-2; sebaceous glands for ERα). For HER2 IHC, the pathway HER2 4-in-1 control slides (Roche Diagnostics) were chosen because they allow the quality of staining for each HER2 score (0, 1+, 2+, 3+) to be assessed. A lymph node was the external positive control used for Bcl-2, in addition to the Bcl-2+ lymphocytes present in the tumor-associated inflammation as internal positive controls.

Scoring of the immunohistochemical stainings was performed by a medical doctor specialist in breast cancer pathology (DL), two certified veterinary pathologists (JA, FN), and a resident in veterinary pathology (ED), with no knowledge of either the clinical outcome or clinicopathologic data. ERα, PR and Ki-67 were scored based on the number of positive nuclei among at least 500 neoplastic cells (manual image analysis using the Image J software, Research Service Branch, National Institute of Health, Bethesda, Maryland, USA). ERα and PR were considered positive if nuclear staining was observed in at least 10% of the neoplastic cells, as previously reported for canine mammary carcinomas [38–40], and human breast cancer [41], as the threshold proposed for ER and PR positivity of feline mammary carcinomas (Allred score equal of higher than 3) [42] was not significantly prognostic in this cohort. The Ki-67 index was used at a 20% threshold to discriminate between highly and poorly proliferative cases among hormone receptor-positive mammary carcinomas [43]. Soares et al. [44] found a Ki-67 cutoff of ≥14% in their cohort of feline mammary carcinomas, however this threshold was not prognostic in our cohort. CK5/6 was considered positive when more than 1% of the tumor cells expressed the marker in their cytoplasm, as previously reported in feline invasive mammary carcinomas [42].

HER2 was scored as score 0 for absence of immunostaining or membrane staining of less than 10% of tumor cells, that is incomplete and is faint/barely perceptible; score 1+ for incomplete and faint/barely perceptible membrane immunostaining in more than 10% of tumor cells; score 2+ for circumferential membrane immunostaining, that is incomplete and/or weak to moderate in more than 10% of tumor cells, or complete and circumferential membrane staining that is intense but within less than 10% of tumor cells; and score 3+ for complete and intense circumferential membrane immunostaining of more than 10% of tumor cells [45]. Carcinomas were considered HER2-positive only for a 3+ IHC score.

The 180 invasive feline mammary carcinomas were classified as luminal (ERα ≥ 10% and/or PR ≥ 10%, any HER2 score) or triple-negative (ERα <  10%, PR <  10%, HER2 score 0 to 2+) according to ER, PR and HER2 expressions [42, 46, 47].

Bcl-2 expression was quantified as the percentage of positive neoplastic cells (with cytoplasmic signal) in at least 500 cancer cells (Bcl-2 index in %), and the prognostic cutoff of 65% was determined by receiver-operating-characteristic curve analysis calculated for 2-year cancer-specific survival.

### Statistical analyses

Statistical analyses were conducted using the MedCalc® statistical software (Ostend, Belgium). Chi-2 tests were used to compare the clinicopathologic characteristics of Bcl-2–positive and Bcl-2–negative FMCs. One-way analysis of variance was used to compare the Bcl-2 index of clinicopathologic subgroups of FMCs. The Kaplan-Meier method and log-rank tests were used in univariate survival analyses, and Cox proportional hazards models for multivariate survival analyses. The results are reported using the Hazard Ratio (HR), its 95% confidence interval (95%-CI), and the *p*-value of each covariate. A *p*-value < 0.05 was considered significant.

## Results

### Cohort description

The cohort comprises 180 female cats with invasive mammary carcinoma, whose main characteristics are listed in Table 2. The mean age at diagnosis was 11.1 ± 2.7 years (range, 4.0–19.3 years). The cats were mainly European (154/180, 85%), 13% were purebred (23/180) with most of them being Siamese (15/23) and 2% were crossbred (3/180).

**Table 2:** Cohort description

Parameters	Categories	N	% of total
Breed	European	154	85.6%
	Other breeds	26	14.4%
Gender	Intact female	112	62.2%
	Neutered female	68	37.8%
History of contraception	Yes	76	42.2%
	No	18	10.0%
	Unknown	86	47.8%
Previous benign mammary lesions	Yes	16	8.9%
	No	164	91.1%
Parity	Nulliparous	15	8.3%
	Multiparous	21	11.7%
	Unknown	144	80.0%
Multicentricity	Multiple FMCs	26	14.4%
	Single FMC	154	85.6%
Pathologic tumor size	pT < 20 mm	85	47.2%
	pT ≥ 20 mm	95	52.8%
Pathologic nodal stage	pN+ (nodal metastasis)	101	56.1%
	pN0 (no)	20	11.1%
	pNX (unknown)	59	32.8%
Distant metastasis	M1 (yes)	8	4.4%
	M0 (no)	64	35.6%
	MX (unknown)	108	60.0%
WHO stage	Stage I	45	25.0%
	Stage II	23	12.8%
	Stage III	104	57.8%
	Stage IV	8	4.4%
WHO Histological type	Adenosquamous	4	2.2%
	Anaplastic	2	1.1%
	Comedocarcinoma	50	27.8%
	Cribriform	54	30.0%
	Mucinous	15	8.3%
	Papillary	7	3.9%
	Solid	27	15.0%
	Tubular	9	5.0%
	Tubulopapillary	12	6.7%
Elston and Ellis histological grading system	Grade I	10	5.5%
	Grade II	82	45.6%
	Grade III	88	48.9%
MMEE grading system	Grade I	62	34.4%
	Grade II	101	56.1%
	Grade III	17	9.4%
Lymphovascular invasion	LVI+	110	61.1%
	LVI–	70	38.9%
Tumor-associated inflammation	Absent to mild	77	42.8%
	Moderate to severe	103	57.2%
Estrogen Receptor (ERα)	ER+ (ER ≥ 10%)	49	27.2%
	ER– (ER < 10%)	131	72.8%
Progesterone Receptor (PR)	PR+ (PR ≥ 10%)	13	7.2%
	PR– (PR < 10%)	167	92.8%
HER2 Score	0	103	57.2%
	1+	59	32.8%
	2+	18	10.0%
	3+	0	0
Ki-67	High Ki-67 (≥ 20%)	169	93.9%
	Low Ki-67 (< 20%)	11	6.1%
CK5/6	CK5/6+ (≥ 1%)	113	62.8%
	CK5/6– (< 1%)	67	37.2%

Twenty-six (14%) of the 180 FMCs were multiple within a given mammary gland or multicentric (affecting more than 1 mammary gland). The mean pathologic tumor size was 18 ± 7 mm (median, 18 mm; range, 4–48 mm) in the 150 cases in which it could be precisely determined; in the 30 remaining cases, tumor size was not measurable on histological sections due to infiltrated tumor margins. Fifty-six percent of the patients (101/180) had a positive pathologic nodal stage (pN+), and 8/180 (4%) had distant metastases (M1) at diagnosis. The 180 FMCs were diagnosed at stage I in 45/180 (25%) cases, stage II in 22/180 (13%), stage III in 104/180 (58%) and stage IV in 8/180 (4%) cats.

Histologically, central necrosis was present in 160/180 cases (89%), squamous differentiation in 81/180 cases (45%), lymphovascular invasion (LVI) in 110/180 cases (61%), and moderate to severe tumor-associated inflammation in 103/180 cases (57%).

Most FMCs expressed the estrogen receptor ERα (88%, 158/180) but only 27% (49/180) were ER-positive at a 10% threshold. The mean ERα index was 10.0 ± 13.3% (median, 5.4%; range, 0–74.2%). The mean PR index was 3.0 ± 11.0% (median, 0%; range, 0–87.8%). Most FMCs were totally devoid of PR expression (80%, 144/180), and only 7% (13/180) were PR-positive (PR index ≥10%). None of the carcinomas expressed HER2 with a 3+ immunohistochemical score. Fifty-seven FMCs (32%) were luminal (ER ≥ 10% and/or PR ≥ 10%, any HER2 score), and 123/180 (68%) were triple-negative (ER–, PR–, HER2–).

The mean Ki-67 index was 45 ± 14% (median, 45%; range, 13–83%). Among the 57 luminal FMCs, 8 were luminal-A (Ki-67 index < 20%), and 49 were luminal-B (Ki-67 ≥ 20%).

Positivity to CK5/6 was present in 113/180 (63%) FMCs, and did not significantly differ between luminal and triple-negative FMCs. Among the 123 triple-negative FMCs, 76 (62%) were positive to CK5/6, and defined as basal-like triple-negative FMCs.

### Frequency of Bcl-2 expression

**Fig. 1:**
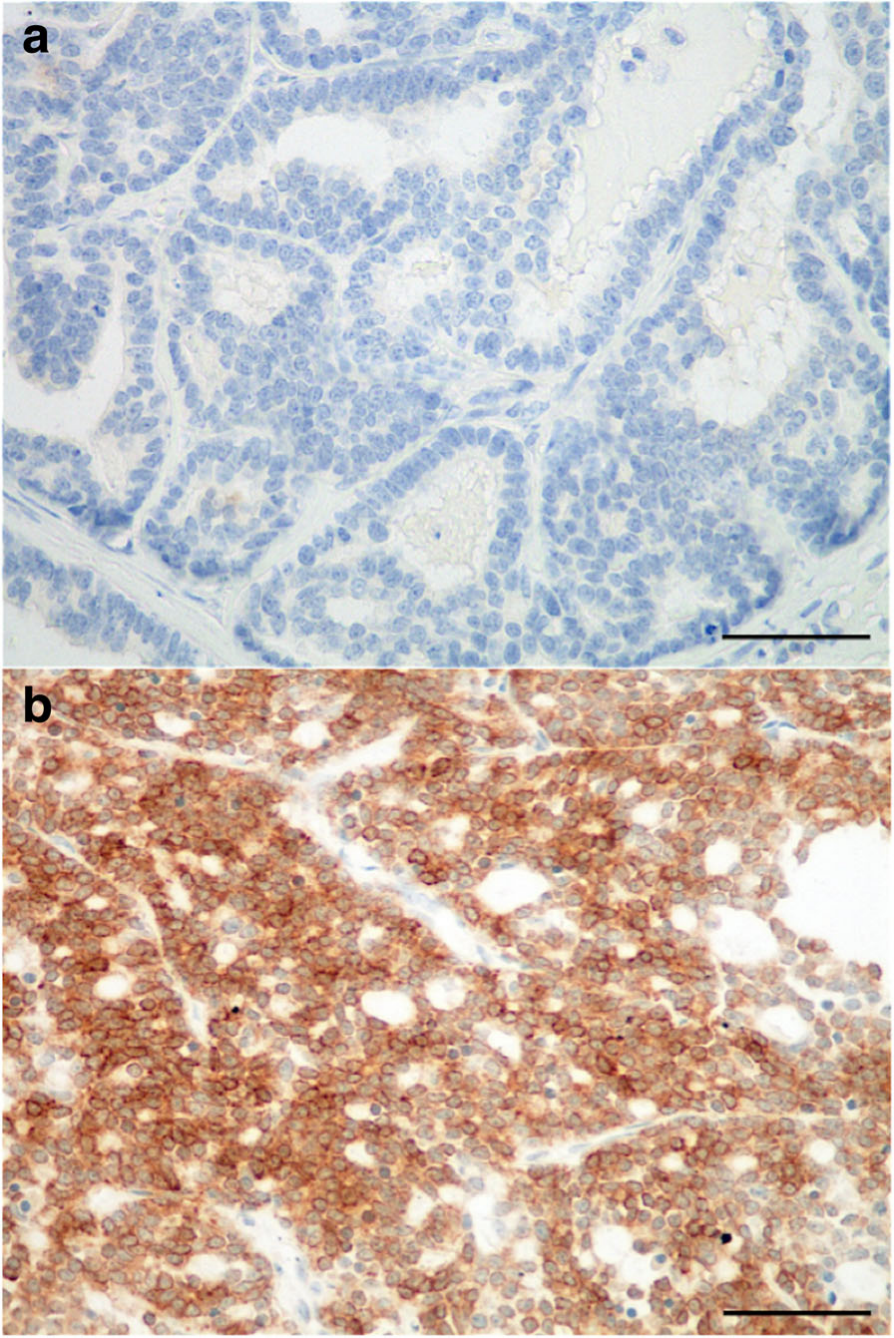
Bcl-2 expression in feline invasive mammary carcinomas determined by immunohistochemistry, depicting a Bcl-2–negative (**a**), and a Bcl-2–positive case (**b**). Peroxidase-DAB revelation system. Original magnification 400x. Bar = 50 μm

Positive immunohistochemical staining to Bcl-2 was cytoplasmic, and observed in neoplastic cells (Fig. 1) as well as some of the lymphocytes in tumor-associated lymphohistiocytic inflammation. The mean Bcl-2 index was 29.4 ± 30.4% (median, 20%; range, 0–95.0%). Most (130/180, 72%) of the FMCs expressed Bcl-2, including 55% (99/180) in at least 10% of the neoplastic cells, and 17% (31/180) in at least 65% of the cancer cells, which was the threshold for positivity with prognostic significance. Using this cutoff, 16% (9/57) of the luminal FMCs, and 18% (22/123) of the 123 triple-negative FMCs were positive to Bcl-2. Bcl-2 positivity was not significantly more common in luminal-A FMCs (2/8 cases) than in luminal-B FMCs (7/49 cases). Among the 76 basal-like triple-negative FMCs, 18% (14/76) were Bcl-2–positive.

### Correlations between Bcl-2 expression and clinical and pathological features

In the global cohort (180 FMCs), Bcl-2 positivity was negatively associated with distant metastasis (*p* = 0.015), squamous differentiation (*p* = 0.002), and the magnitude of tumor-associated inflammation (*p* = 0.038). There was no significant association between Bcl-2 positivity and the pathologic tumor size, pathologic nodal stage, histological grade, Ki-67 proliferation index, ER, PR, HER2 expression, and the immunophenotype.

In the 57 luminal FMCs, as well as in the 123 triple-negative FMCs, there was no significant association between Bcl-2 positivity and the pathologic tumor size, pathologic nodal stage, histological grade, and Ki-67 index. In the 76 triple-negative basal-like FMCs, Bcl-2 positivity was significantly negatively correlated with the severity of peritumoral lymphohistiocytic inflammation (*p* = 0.005).

### Bcl-2 prognostic value in the global cohort (180 FMCs)

**Fig. 2:**
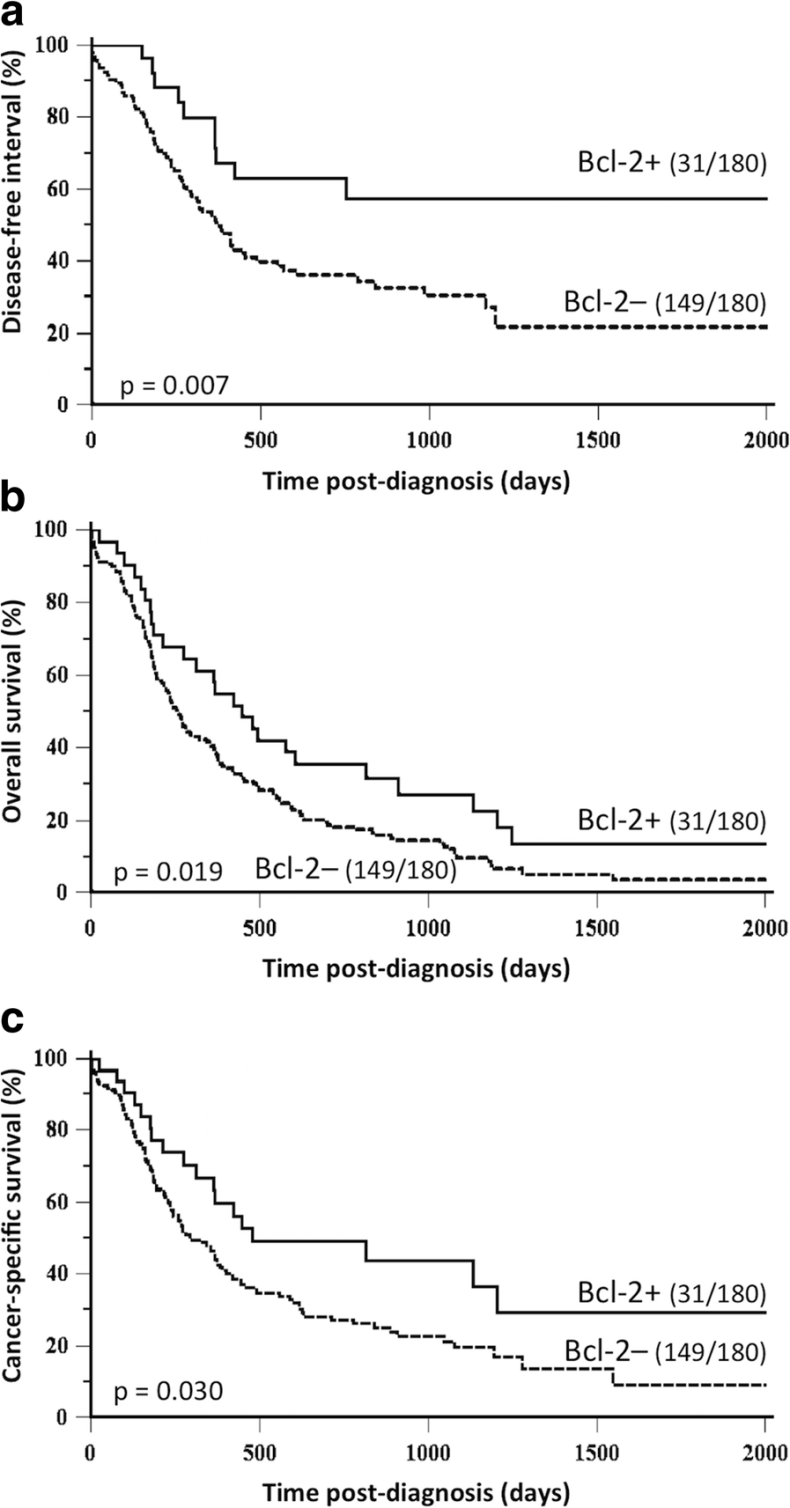
Kaplan-Meier survival curves of 180 cats according to Bcl-2 expression (at 65% threshold) in their invasive mammary carcinomas. **a** Disease-free interval. **b** Overall survival. **c** Cancer-specific survival

By univariate survival analysis, Bcl-2 positivity was associated with a favorable outcome in the global cohort, in terms of disease-free interval (HR = 0.44, 95% CI: 0.27–0.71; *p* = 0.007; Fig.2a), overall survival (HR = 0.61, 95%-CI: 0.43–0.88; *p* = 0.019; Fig. 2b), and cancer-specific survival (HR = 0.59, 95% CI: 0.39–0.90; *p* = 0.030; Fig. 2c). In the first 2 years post-diagnosis, 64% of cats had experienced locoregional recurrence and/or distant metastasis in the Bcl-2–negative group, compared to 37% in the Bcl-2–positive group. The median overall survival was 449 days (14.7 months) for female cats with a Bcl-2–positive FMC, but only 259 days (8.5 months) for female cats with a Bcl-2–negative FMC. At 2 years post-diagnosis, 73% of cats with a Bcl-2–negative FMC had died from cancer, compared to 51% of cats with a Bcl-2–positive FMC.

By multivariate survival analysis using Cox proportional hazards regression, Bcl-2 positivity was associated with a longer disease-free interval (HR = 0.57, 95%-CI: 0.34–0.96; *p* = 0.034) independently of the pathologic tumor size (pT ≥ 20 mm: HR = 1.52, 95%-CI: 1.04–2.21; *p* = 0.029), and positive nodal stage (pN+ compared to pN0: HR = 2.36, 95%-CI: 1.27–4.41; *p* = 0.007; Table 3).

**Table 3:** Prognostic value of Bcl-2 expression estimated by multivariate analysis in the global cohort of 180 FMCs

	Categories	p	HR	95%-CI
Disease-free interval				
Bcl-2 expression	Bcl-2+ versus Bcl-2–	0.034	0.57	0.34–0.96
Pathologic tumor size	pT ≥ 20 mm versus < 20 mm	0.029	1.52	1.04–2.21
Pathologic nodal stage	pNX versus pN0	0.109	1.71	0.89–3.28
	pN+ versus pN0	0.007	2.36	1.27–4.41
Overall survival				
Bcl-2 expression	Bcl-2+ versus Bcl-2–	0.015	0.58	0.37–0.90
Pathologic tumor size	pT ≥ 20 mm versus < 20 mm	0.002	1.64	1.20–2.25
Pathologic nodal stage	pNX versus pN0	0.252	1.38	0.80–2.36
	pN+ versus pN0	0.003	2.15	1.30–3.55
Distant metastasis	MX versus M0	0.083	0.74	0.53–1.04
	M1 versus M0	0.034	2.28	1.07–4.87
Tumor-associated inflammation	Absence to mild versus moderate to severe	0.034	0.71	0.51–0.97
Cancer-specific survival				
Bcl-2 expression	Bcl-2+ versus Bcl-2–	0.008	0.50	0.30–0.84
Pathologic tumor size	pT ≥ 20 mm versus < 20 mm	0.002	1.78	1.24–2.55
Pathologic nodal stage	pNX versus pN0	0.296	1.42	0.74–2.75
	pN+ versus pN0	0.002	2.63	1.44–4.80
Distant metastasis	MX versus M0	0.026	0.65	0.44–0.95
	M1 versus M0	0.022	2.47	1.14–5.32

The favorable prognostic value of Bcl-2 positivity was also significant in overall survival (HR = 0.58, 95%-CI: 0.37–0.90; *p* = 0.015) with the pathologic tumor size, pathologic nodal stage, distant metastasis, and tumor-associated inflammation as the covariates of the model (Table 3).

The probability of cancer-related death in this cohort of female cats with invasive mammary carcinoma could be predicted with Bcl-2 expression and the 3 parameters of cancer stage (tumor size, nodal stage, and distant metastasis; Table 3). In this model, Bcl-2 positivity of feline invasive mammary carcinomas was associated with a 2-fold decrease in the risk of cancer-related death (HR = 0.50, 95%-CI: 0.30–0.84; *p* = 0.008).

### Bcl-2 prognostic value in cats with luminal FMC

**Fig. 3:**
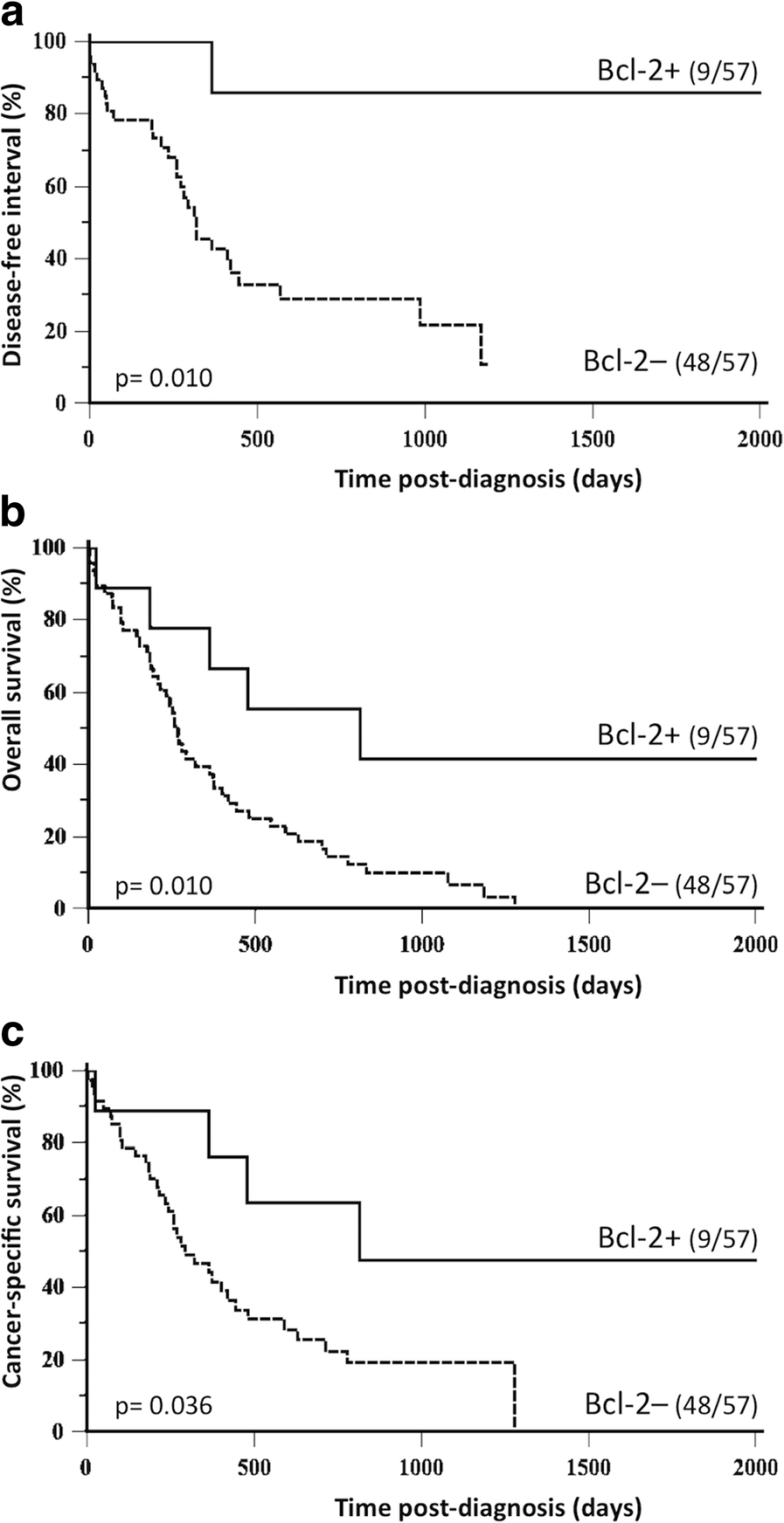
Kaplan-Meier survival curves of 57 cats with luminal-FMCs according to Bcl-2 expression (at 65% threshold for positivity). **a** Disease-free interval. **b** Overall survival. **c** Cancer-specific survival

By univariate survival analysis, Bcl-2 positivity was associated with a good outcome in cats with luminal FMC, in terms of disease-free interval (HR = 0.20, 95% CI: 0.09–0.46; *p* = 0.010; Fig. 3a), overall survival (HR = 0.36, 95%-CI: 0.20–0.67; *p* = 0.010; Fig. 3b), and cancer-specific survival (HR = 0.40, 95% CI: 0.20–0.82; *p* = 0.036; Fig. 3c).

By multivariate survival analysis, Bcl-2 positivity was associated with a longer disease-free interval (HR = 0.09, 95%-CI: 0.01–0.64; *p* = 0.017), a longer overall survival (HR = 0.28, 95%-CI: 0.11–0.72; *p* = 0.008), and a lower risk of cancer-related death (HR = 0.31, 95%-CI: 0.11–0.89; *p* = 0.031), as depicted in Table 4, with in each model, the pathologic tumor size and tumor-associated inflammation as the covariates. These results indicate that Bcl-2 expression, tumor size, and cancer-associated lymphohistiocytic inflammation were the 3 significant and independent predictors of the outcome of female cats with ER+ and/or PR+ mammary carcinoma in this cohort.

**Table 4:** Prognostic value of Bcl-2 expression estimated by multivariate analysis in 57 luminal FMCs

	Categories	p	HR	95%-CI
Disease-free interval				
Bcl-2 expression	Bcl-2+ versus Bcl-2–	0.017	0.09	0.01–0.64
Pathologic tumor size	pT ≥ 20 mm versus < 20 mm	0.047	2.23	1.02–4.91
Tumor-associated inflammation	Absence to mild versus moderate to severe	0.033	0.40	0.18–0.92
Overall survival				
Bcl-2 expression	Bcl-2+ versus Bcl-2–	0.008	0.28	0.11–0.72
Pathologic tumor size	pT ≥ 20 mm versus < 20 mm	0.014	2.07	1.16–3.70
Tumor-associated inflammation	Absence to mild versus moderate to severe	0.012	0.44	0.23–0.84
Cancer-specific survival				
Bcl-2 expression	Bcl-2+ versus Bcl-2–	0.031	0.31	0.11–0.89
Pathologic tumor size	pT ≥ 20 mm versus < 20 mm	0.026	2.10	1.09–4.02
Tumor-associated inflammation	Absence to mild versus moderate to severe	0.022	0.43	0.21–0.88

### Bcl-2 prognostic value in cats with triple-negative FMC

There was no significant association between Bcl-2 expression and the outcome of the 123 female cats with triple-negative FMC. However, there was a non-significant trend for Bcl-2 positivity to be associated with favorable disease-free interval (*p* = 0.06), overall survival (*p* = 0.07), and specific survival (*p* = 0.09) in the subcohort of 76 triple-negative basal-like FMCs. By multivariate survival analysis, Bcl-2 positivity of triple-negative basal-like FMCs was associated with a longer overall survival (HR = 0.53, 95%-CI: 0.28–0.99; *p* = 0.047) independently of the WHO feline staging system (Table 5), and a lower risk of cancer-related death (HR = 0.45, 95%-CI: 0.21–0.99; *p* = 0.047) independently of the pathologic nodal stage (Table 5).

**Table 5:** Prognostic value of Bcl-2 expression estimated by multivariate analysis in 76 triple-negative basal-like FMCs

	Categories	p	HR	95%-CI
Overall survival				
Bcl-2 expression	Bcl-2+ versus Bcl-2–	0.047	0.53	0.28–0.99
WHO stage	Stages I–II versus III–IV	0.004	2.13	1.28–3.54
Cancer-specific survival				
Bcl-2 expression	Bcl-2+ versus Bcl-2–	0.047	0.45	0.21–0.99
Pathologic nodal stage	pNX versus pN0	0.158	2.22	0.74–6.72
	pN+ versus pN0	0.004	4.66	1.63–13.34

## Discussion

The objective of our study was to investigate Bcl-2 expression in a large cohort of feline mammary carcinomas, and to assess its relationship with other clinical and pathological features. We also aimed at investigating the prognostic significance of Bcl-2 expression in FMCs in order to evaluate its significance in the feline patient, and to compare with previous studies in human breast cancer. The results showed that Bcl-2 was a powerful prognostic factor that was independent of cancer stage. When compared to the Bcl-2 negative group of FMCs, the Bcl-2 positive group showed a more favorable outcome in terms of disease-free interval, overall survival and specific survival, both in luminal and basal-like triple-negative FMCs.

In our study, Bcl-2 was frequently expressed in feline mammary carcinomas, as 72% of the cases expressed some kind of staining in the cytoplasm of tumor cells. Using the most significant threshold for positivity of 65%, 17% of FMCs (31/180) were positive for Bcl-2. Our results are relatively close to those reported in human breast cancer studies in terms of frequency of Bcl-2 expression: at 10% threshold for positivity, Bcl-2 is expressed in 68.2% of the 7230 human invasive mammary carcinomas according to the large-scale study by Hwang et al. [9] in comparison to 52.2% in our study. But the frequency of Bcl-2 expression varies enormously depending on the authors: from 35% of the 346 metastatic breast cancers studied by Chang et al. [48] to 74.9% of the 250 cases described by Hellemans et al. [49].

Bcl-2 expression in FMCs was previously reported in another study in the literature. According to Madewell et al. [31], Bcl-2 was also frequently expressed in FMCs, it concerned 13 out of the 15 invasive mammary carcinomas, (i.e., 86.7% of the studied carcinomas) with Bcl-2-positive carcinomas containing 20–80% of immunolabeled cells.

In our study and especially in the global cohort, Bcl-2 positivity was inversely associated with aggressive features, such as distant metastasis at diagnosis. These results are in agreement with the studies published on human breast cancer, where Bcl-2 positivity has been associated with favorable clinicopathologic features, such as smaller tumor size [9, 12, 50–56], and negative nodal status [12, 18, 51, 52], in both triple-negative and non-triple-negative breast cancer [57], although in the present study, Bcl-2 positivity of FMCs was not significantly correlated with the pathologic tumor size and nodal stage.

There are other differences between feline and human invasive mammary carcinomas with respect to Bcl-2 expression, as there was no significant association between Bcl-2 and the histological grade, ER, PR, Ki-67, or the luminal phenotype in the present study. Conversely, in human breast cancer, Bcl-2 positivity is associated with a lower histological grade according to numerous publications [7, 9, 18, 51, 52, 58–63], including in locally advanced breast cancers [51, 53]. A consistent and strong positive correlation between Bcl-2 positivity and hormone receptor expression is reported in breast cancer, and concerns both ER and PR [7, 9, 12, 19, 49, 51, 52, 54, 55, 58–60]. Bcl-2 positivity of breast cancers has been associated with a lower Ki-67 proliferation index [7, 12, 16, 50, 60], with the luminal phenotype [7], and especially the luminal-A phenotype [64]. Indeed, *BCL2* is upregulated by estrogens in breast cancer cells [65], explaining why Bcl2-positive expression in breast cancer may be considered a sign of ER functional activity [7]. By comparison, luminal FMCs were relatively rare in this cohort (57/180, 32% of the cases) compared to triple-negative FMCs, and Bcl-2 positivity was not associated with the luminal or triple-negative phenotype. This suggests an overrepresentation in the feline species of invasive mammary carcinomas in which the ER pathway is poorly functional, and in which Bcl-2 expression does not proceed from estrogen exposure, but rather from other mechanisms still to be determined.

Our results show that Bcl-2 positivity in FMCs is associated with a better outcome by univariate and multivariate analysis in terms of disease-free interval, overall survival and cancer-specific survival in the global cohort. In human breast cancer, Bcl-2 positivity is also associated with a decreased risk of cancer progression and death. For example, Hwang et al. [9] showed in a series of 7230 primary breast cancer patients, that Bcl-2 positivity was associated with better overall survival (HR, 0.42; 95%-CI, 0.42–0.71; *p* < 0.001), independently of patient age at diagnosis, the histologic grade, tumor size and nodal stage, hormone receptor and HER2 expression, and lymphovascular invasion. Dawson et al. [11] showed that positivity to Bcl-2 was associated with a longer survival independently of tumor size, histologic grade, hormone receptor status, and HER2 overexpression. Bcl-2 positivity has also been associated with longer disease-free survival [5–10, 60], longer overall survival [5, 6, 8, 10, 11, 13, 49, 66–68], and lower risk of cancer-related death [14, 52, 69] in other reports, especially in luminal breast cancers [7, 12, 18].

In the 57 luminal FMCs, Bcl-2 positivity was also associated with better outcome in terms of disease-free interval, overall survival and specific survival, independently of the pathologic tumor size and tumor-associated inflammation. Many studies in human breast cancer have reported the protective effect of Bcl-2 positivity in the luminal phenotype. Kim et al. [17] showed by univariate analysis in their study of 159 patients with luminal (ER+ and/or PR+) breast carcinoma, that Bcl-2 positivity (at 33% cutoff for positivity) was one of the 4 prognostic factors associated with progression-free survival, the others being patient age at diagnosis (at a threshold of 35-years), tumor size (at a threshold of 2-cm), and nodal stage (positive / negative). By multivariate analysis, only Bcl-2 and the nodal stage retained their independent prognostic value [17].

We also investigated the prognostic significance of Bcl-2 in the triple-negative basal-like (ER, PR, and HER2 negative, and CK5/6 positive) subtype of FMCs. Among the 76 basal-like triple-negative FMCs, 18% (14/76) were Bcl-2–positive, and were associated with a better outcome in terms of overall survival, and specific survival, independently of the pathologic nodal stage or the WHO cancer stage. These results indicate that there exists a subgroup of triple-negative basal-like FMCs, identified in this cohort by CK5/6 positivity, in which Bcl-2 expression exerts a favorable prognostic influence. In human breast cancer, the prognostic significance of Bcl-2 positivity in triple-negative carcinomas is controversial. Abdel-Fatah et al. [20] reported that Bcl-2 negativity in triple-negative breast cancer was associated with a 2-fold increased risk of death (HR = 1.71; 95%-CI 1.21–2.41; *p* = 0.002) and recurrence (HR = 1.79; 95%-CI 1.34–2.38; *p* = 0.0005); in multivariate survival analysis, Bcl-2 negativity was also associated with worse cancer-specific survival and progression-free survival independently of the nodal stage. Other studies however showed that Bcl-2 positivity is associated with a worse prognosis in triple-negative breast cancer [21, 58, 70], and finally some authors did not find any prognostic significance of Bcl-2 in triple-negative breast cancer [12]. These discrepant findings might result from the heterogeneity of triple-negative breast cancers (TNBCs), which comprise at least “luminal-Androgen Receptor” and “basal-like” subtypes according to gene expression studies [71]. Bcl-2 may have different roles in different TNBC subtypes, as it was shown for instance that Bcl-2 positivity is more common in non basal-like than in basal-like TNBCs [72], and *BCL2* gene expression is highest in the “mesenchymal stem-like” molecular subtype of TNBC defined by Lehmann et al. [71].

The biological mechanisms underlying the prognostic role of Bcl-2 in human breast cancer remain mysterious and largely uncertain. Bcl-2 is well known as an anti-apoptotic oncogene in lymphomas [73], and this anti-apoptotic function may explain why Bcl-2 positivity has been associated with the chemoresistance [63, 74, 75] and radioresistance [76] of breast cancer cells. However, the paradoxical antiproliferative function of this tumor suppressor gene has been described in multiple human cancer cell lines and murine mammary carcinoma models [15, 77]. Bcl-2 may thus be tumor-suppressive and at the same time oncogenic in specific cell types or under specific circumstances. Of importance, the Bcl-2 protein does not act alone in regulating mitochondrial outer membrane permeabilization, but rather in interaction with other Bcl-2 family members, which are not only involved in regulating the intrinsic pathway of apoptosis, but also calcium homeostasis, autophagy, and possibly DNA repair in cancer cells [78]. Thus concomitant evaluations of the expression of several Bcl-2 family members may be required to understand their potential effects in feline mammary carcinoma development and prognosis.

## Conclusion

This is the first study that investigates the prognostic significance of Bcl-2 in feline mammary carcinomas. We showed, as described in human breast cancer, that Bcl-2 positivity was associated with a favorable clinical outcome in terms of disease-free interval, overall survival, and specific survival in feline invasive mammary carcinomas, which suggests that Bcl-2 may have similar roles in human and feline mammary carcinomas. The cat appears to be a relevant naturally-occurring model for Bcl-2 overexpression in both luminal and triple-negative basal-like carcinomas, opening the way for possible translational perspectives, especially for evaluating in cats new therapeutic agents targeting Bcl-2 for chemoresistant luminal or triple-negative breast cancers.

## Abbreviations

Bcl-2: B cell lymphoma/leukemia-2
ER: Estrogen Receptor alpha
FMC: Feline invasive Mammary Carcinoma
HER2: Human Epidermal growth factor Receptor-2
HIER: Heat-induced epitope retrieval
HR: Hazard ratio
LVI: Lymphovascular invasion
MMEE: Mitotic-modified Elston and Ellis grading system
pN: pathologic nodal stage
PR: Progesterone Receptor
pT: pathologic tumor size
TNBC: Triple-negative breast cancer
CK5/6: Cytokeratins 5 and 6

## References

[1] Tsujimoto Y, Croce CM (1986). Analysis of the structure, transcripts, and protein products of bcl-2, the gene involved in human follicular lymphoma. Proc Natl Acad Sci U S A.

[2] Adams JM, Cory S (1998). The Bcl-2 protein family: arbiters of cell survival. Science.

[3] Frenzel A, Grespi F, Chmelewskij W, Villunger A (2009). Bcl2 family proteins in carcinogenesis and the treatment of cancer. Apoptosis.

[4] Reed JC (1994). Bcl-2 and the regulation of programmed cell death. J Cell Biol.

[5] Callagy GM, Webber MJ, Pharoah PD, Caldas C (2008). Meta-analysis confirms BCL2 is an independent prognostic marker in breast cancer. BMC Cancer.

[6] Berardo MD, Elledge RM, de Moor C, Clark GM, Osborne CK, Allred DC (1998). Bcl-2 and apoptosis in lymph node positive breast carcinoma. Cancer.

[7] Eom YH, Kim HS, Lee A, Song BJ, Chae BJ (2016). BCL2 as a subtype-specific prognostic marker for breast Cancer. J Breast Cancer.

[8] Gasparini G, Barbareschi M, Doglioni C, Palma PD, Mauri FA, Boracchi P (1995). Expression of bcl-2 protein predicts efficacy of adjuvant treatments in operable node-positive breast cancer. Clin Cancer Res.

[9] Hwang KT, Woo JW, Shin HC, Kim HS, Ahn SK, Moon HG (2012). Prognostic influence of BCL2 expression in breast cancer. Int J Cancer.

[10] Silvestrini R, Veneroni S, Daidone MG, Benini E, Boracchi P, Mezzetti M (1994). The Bcl-2 protein: a prognostic indicator strongly related to p53 protein in lymph node-negative breast cancer patients. J Natl Cancer Inst.

[11] Dawson SJ, Makretsvov N, Blows FM, Driver KE, Provenzano E, Le Quesne J (2010). BCL2 in breast cancer: a favourable prognostic marker across molecular subtypes and independent of adjuvant therapy received. Br J Cancer.

[12] Hwang K, Han W, Kim J, Moon H, Oh S, Seon Song Y, Kim Y (2017). Prognostic influence of BCL2 on molecular subtypes of breast Cancer. J Breast Cancer.

[13] McCallum M, Baker C, Gillespie K, Cohen B, Stewart H, Leonard R (2004). A prognostic index for operable, node-negative breast cancer. Br J Cancer.

[14] Lê MG, Mathieu MC, Douc-Rasy S, Le Bihan ML, Abd El All H, Spielmann M (1999). c-myc, p53 and bcl-2, apoptosis-related genes in infiltrating breast carcinomas: evidence of a link between bcl-2 protein over-expression and a lower risk of metastasis and death in operable patients. Int J Cancer.

[15] Zinkel S, Gross A, Yang E (2006). BCL2 family in DNA damage and cell cycle control. Cell Death Differ.

[16] Ali HR, Dawson SJ, Blows FM, Provenzano E, Leung S, Nielsen T, Pharoah PD, Caldas C (2012). A Ki67/BCL2 index based on immunohistochemistry is highly prognostic in ER-positive breast cancer. J Pathol.

[17] Kim HS, Park I, Cho HJ, Gwak G, Yang K, Bae BN (2012). Analysis of the potent prognostic factors in luminal-type breast cancer. J Breast Cancer.

[18] Larsen MS, Bjerre K, Giobbie-Hurder A, Lænkholm AV, Henriksen KL, Ejlertsen B, Lykkesfeldt AE, Rasmussen BB (2012). Prognostic value of Bcl-2 in two independent populations of estrogen receptor positive breast cancer patients treated with adjuvant endocrine therapy. Acta Oncol.

[19] Seong MK, Lee JY, Byeon J, Sohn YJ, Seol H, Lee JK (2015). Bcl-2 is a highly significant prognostic marker of hormone-receptor-positive, human epidermal growth factor receptor-2-negative breast cancer. Breast Cancer Res Treat.

[20] Abdel-Fatah TM, Perry C, Dickinson P, Ball G, Moseley P, Madhusudan S (2013). Bcl2 is an independent prognostic marker of triple negative breast cancer (TNBC) and predicts response to anthracycline combination (ATC) chemotherapy (CT) in adjuvant and neoadjuvant settings. Ann Oncol.

[21] Honma N, Horii R, Ito Y, Saji S, Younes M, Iwase T (2015). Differences in clinical importance of Bcl-2 in breast cancer according to hormone receptors status or adjuvant endocrine therapy. BMC Cancer.

[22] Hayes AA, Mooney S (1985). Feline mammary tumors. Vet Clin North Am Small Anim Pract.

[23] Misdorp W, Weijer K (1980). Animal model of human disease: breast cancer. Am J Pathol.

[24] Gimenez F, Hecht S, Craig LE, Legendre AM (2010). Early detection, aggressive therapy optimizing the management of feline mammary masses. J Feline Med Surg..

[25] Castagnaro M, Casalone C, Bozzetta E, De Maria R, Biolatti B, Caramelli M (1998). Tumour grading and the one-year post-surgical prognosis in feline mammary carcinomas. J Comp Pathol.

[26] Castagnaro M, Casalone C, Ru G, Nervi GC, Bozzetta E, Caramelli M (1998). Argyrophilic nucleolar organiser regions (AgNORs) count as indicator of post-surgical prognosis in feline mammary carcinomas. Res Vet Sci.

[27] Castagnaro M, De Maria R, Bozzetta E, Ru G, Casalone C, Biolatti B (1998). Ki-67 index as indicator of the post-surgical prognosis in feline mammary carcinomas. Res Vet Sci.

[28] Ito T, Kadosawa T, Mochizuki M, Matsunaga S, Nishimura R, Sasaki N (1996). Prognosis of malignant mammary tumor in 53 cats. J Vet Med Sci.

[29] Millanta F, Calandrella M, Citi S, Della Santa D, Poli A (2005). Overexpression of HER-2 in feline invasive mammary carcinomas: an immunohistochemical survey and evaluation of its prognostic potential. Vet Pathol.

[30] Seixas F, Palmeira C, Pires MA, Bento MJ, Lopes C (2011). Grade is an independent prognostic factor for feline mammary carcinomas: a clinicopathological and survival analysis. Vet J.

[31] Madewell BR, Gandour-Edwards R, Edwards BF, Walls JE, Griffey SM (1999). Topographic distribution of bcl-2 protein in feline tissues in health and neoplasia. Vet Pathol.

[32] Moriya T, Kanomata N, Kozuka Y, Fukumoto M, Iwachido N, Hata S (2009). Usefulness of immunohistochemistry for differential diagnosis between benign and malignant breast lesions. Breast Cancer.

[33] Shamloula MM, El-Shorbagy SH, Saied EME (2007). P63 and cytokeratin8/18 expression in breast, atypical ductal hyperplasia, ductal carcinoma in situ and invasive duct carcinoma. J Egypt Natl Canc Inst.

[34] Elston CW, Ellis IO (1991). Pathological prognostic factors in breast cancer: I. the value of histological grade in breast cancer: experience from a large study with long-term follow-up. Histopathology.

[35] Mills SW, Musil KM, Davies JL, Hendrick S, Duncan C, Jackson ML (2015). Prognostic value of histologic grading for feline mammary carcinoma: a retrospective survival analysis. Vet Pathol.

[36] Morris J (2013). Mammary tumors in the cat: size matters, so early intervention saves lives. J Feline Med Surg.

[37] Owen LN, Owen LN, World Health Organization (1980). Veterinary public health unit & WHO collaborating Center for Comparative Oncology. TNM classification of Tumours in domestic animals.

[38] Gama A, Alves A, Schmitt F (2008). Identification of molecular phenotypes in canine mammary carcinomas with clinical implications: application of the human classification. Virchows Arch.

[39] Guil-Luna S, Sánchez-Céspedes R, Millán Y, De Andrés FJ, Rollón E, Domingo V (2011). Aglepristone decreases proliferation in progesterone receptor-positive canine mammary carcinomas. J Vet Intern Med.

[40] Nguyen F, Peña L, Ibisch C, Loussouarn D, Gama A, Rieder N (2018). Canine invasive mammary carcinomas as models of human breast cancer. Part 1: natural history and prognostic factors. Breast Cancer Res Treat.

[41] Yi M, Huo L, Koenig KB, Mittendorf EA, Meric-Bernstam F, Kuerer HM (2014). Which threshold for ER positivity? A retrospective study based on 9639 patients. Ann Oncol.

[42] Soares M, Correia J, Peleteiro MC, Ferreira F (2016). St Gallen molecular subtypes in feline mammary carcinoma and paired metastases-disease progression and clinical implications from a 3-year follow-up study. Tumour Biol.

[43] Ono M, Tsuda H, Yunokawa M, Yonemori K, Shimizu C, Tamura K (2015). Prognostic impact of Ki-67 labeling indices with 3 different cutoff values, histological grade, and nuclear grade in hormone-receptor-positive, HER2-negative, node-negative invasive breast cancers. Breast Cancer..

[44] Soares M, Ribeiro R, Carvalho S, Peleteiro M, Correia J, Ferreira F (2016). Ki-67 as a prognostic factor in feline mammary carcinoma: what is the optimal cutoff value?. Vet Pathol.

[45] Wolff AC, Hammond ME, Hicks DG, Dowsett M, McShane LM, Allison KH (2014). Recommendations for human epidermal growth factor receptor 2 testing in breast cancer: American Society of Clinical Oncology/College of American Pathologists clinical practice guideline update. Arch Pathol Lab Med.

[46] Brunetti B, Asproni P, Beha G, Muscatello LV, Millanta F, Poli A (2013). Molecular phenotype in mammary tumours of queens: correlation between primary tumour and lymph node metastasis. J Comp Pathol.

[47] Wiese DA, Thaiwong T, Yuzbasiyan-Gurkan V, Kiupel M (2013). Feline mammary basal-like adenocarcinomas: a potential model for human triple-negative breast cancer (TNBC) with basal-like subtype. BMC Cancer.

[48] Chang J, Clark GM, Allred DC, Mohsin S, Chamness G, Elledge RM (2003). Survival of patients with metastatic breast carcinoma. Cancer.

[49] Hellemans P, Van Dam PA, Weyler J, Van Oosterom AT, Buytaert P, Van Marck E (1995). Prognostic value of bcl-2 expression in invasive breast cancer. Br J Cancer.

[50] Bottini A, Berruti A, Bersiga A, Brizzi MP, Brunelli A, Gorzegno G, DiMarco B, Aguggini S, Bolsi G, Cirillo F, Filippini L, Betri E, Bertoli G, Alquati P, Dogliotti L (2000). p53 but not bcl-2 immunostaining is predictive of poor clinical complete response to primary chemotherapy in breast cancer patients. Clin Cancer Res.

[51] Chen HH, Su WC, Guo HR, Chang TW, Lee WY (2002). p53 and c-erbB-2 but not bcl-2 are predictive of metastasis-free survival in breast cancer patients receiving post-mastectomy adjuvant radiotherapy in Taiwan. Jpn J Clin Oncol.

[52] Ermiah E, Buhmeida A, Khaled BR, Abdalla F, Salem N, Pyrhönen S (2013). Prognostic value of bcl-2 expression among women with breast cancer in Libya. Tumour Biol.

[53] Kyndi M, Sørensen FB, Knudsen H, Alsner J, Overgaard M, Nielsen HM, Overgaard J (2008). Impact of BCL2 and p53 on postmastectomy radiotherapy response in high-risk breast cancer. A subgroup analysis of DBCG82 b&c. Acta Oncol.

[54] Villar E, Redondo M, Rodrigo I, García J, Avila E, Matilla A (2001). Bcl-2 expression and apoptosis in primary and metastatic breast carcinomas. Tumour Biol.

[55] Zhang GJ, Kimijima I, Abe R, Kanno M, Katagata N, Hara K (1997). Correlation between the expression of apoptosis-related bcl-2 and p53 oncoproteins and the carcinogenesis and progression of breast carcinomas. Clin Cancer Res.

[56] Zhang GJ, Tsuda H, Adachi I, Fukutomi T, Yamamoto H, Hirohashi S (1997). Prognostic indicators for breast cancer patients with one to three regional lymph node metastases, with special reference to alterations in expression levels of bcl-2, p53 and c-erbB-2 proteins. Jpn J Clin Oncol.

[57] Abd El-Hafez A, Ael-A SM, Elesawy BH (2013). Different prognostic factors correlate with Bcl-2 expression among triple negative and non-triple negative breast cancers. Asian Pac J Cancer Prev.

[58] Lee JY, Kim HA, Kim EK, Yang HM, Kim KI, Lee JI (2011). Different prognostic significance of Bcl-2 based on Cancer molecular subtype. J Breast Cancer.

[59] Bhargava V, Kell DL, van de Rijn M, Warnke RA (1994). Bcl-2 immunoreactivity in breast carcinoma correlates with hormone receptor positivity. Am J Pathol.

[60] Biesaga B, Niemiec J, Ziobro M (2014). BCL-2, topoisomerase IIα, microvessel density and prognosis of early advanced breast cancer patients after adjuvant anthracycline-based chemotherapy. J Cancer Res Clin Oncol.

[61] Joensuu H, Pylkkänen L, Toikkanen S (1994). Bcl-2 protein expression and long-term survival in breast cancer. Am J Pathol.

[62] Lee HD, Koo JY, Jung WH (1997). Correlations of Bcl-2 expression with clinicopathological features in breast cancer. Yonsei Med J.

[63] Yu B, Sun X, Shen HY, Gao F, Fan YM, Sun ZJ (2010). Expression of the apoptosis-related genes BCL-2 and BAD in human breast carcinoma and their associated relationship with chemosensitivity. J Exp Clin Cancer Res.

[64] Escórcio-Dourado CS, Martins LM, Simplício-Revoredo CM, Sampaio FA, Tavares CB, da Silva-Sampaio JP (2017). Bcl-2 antigen expression in luminal a and triple-negative breast cancer. Med Oncol.

[65] Leung LK, Wang TT (1999). Paradoxical regulation of Bcl-2 family proteins by 17beta-oestradiol in human breast cancer cells MCF-7. Br J Cancer.

[66] Bhatavdekar JM, Patel DD, Shah NG, Vora HH, Suthar TP, Chikhlikar PR (2000). Prognostic significance of immunohistochemically localized biomarkers in stage II and stage III breast cancer: a multivariate analysis. Ann Surg Oncol.

[67] Callagy GM, Pharoah PD, Pinder SE, Hsu FD, Nielsen TO, Ragaz J (2006). Bcl-2 is a prognostic marker in breast cancer independently of the Nottingham prognostic index. Clin Cancer Res.

[68] Castiglione F, Sarotto I, Fontana V, Destefanis M, Venturino A, Ferro S (1999). Bcl2, p53 and clinical outcome in a series of 138 operable breast cancer patients. Anticancer Res.

[69] Abdel-Fatah TM, Powe DG, Ball G, Lopez-Garcia MA, Habashy HO, Green AR (2010). Proposal for a modified grading system based on mitotic index and Bcl2 provides objective determination of clinical outcome for patients with breast cancer. J Pathol.

[70] Tawfik K, Kimler BF, Davis MK, Fan F, Tawfik O (2012). Prognostic significance of Bcl-2 in invasive mammary carcinomas: a comparative clinicopathologic study between “triple-negative” and non-“triple-negative” tumors. Hum Pathol.

[71] Lehmann BD, Bauer JA, Chen X, Sanders ME, Chakravarthy AB, Shyr Y, Pietenpol JA (2011). Identification of human triple-negative breast cancer subtypes and preclinical models for selection of targeted therapies. J Clin Invest.

[72] Bidard FC, Conforti R, Boulet T, Michiels S, Delaloge S, André F (2007). Does triple-negative phenotype accurately identify basal-like tumour? An immunohistochemical analysis based on 143 ‘triple-negative’ breast cancers. Ann Oncol.

[73] McDonnell TJ, Korsmeyer SJ (1991). Progression from lymphoid hyperplasia to high-grade malignant lymphoma in mice transgenic for the t(14;18). Nature.

[74] Davis JM, Navolanic PM, Weinstein-Oppenheimer CR, Steelman LS, Hu W, Konopleva M, Blagosklonny MV, McCubrey JA (2003). Raf-1 and Bcl-2 induce distinct and common pathways that contribute to breast cancer drug resistance. Clin Cancer Res.

[75] Kutuk O, Letai A (2008). Alteration of the mitochondrial apoptotic pathway is key to acquired paclitaxel resistance and can be reversed by ABT-737. Cancer Res.

[76] Li JY, Li YY, Jin W, Yang Q, Shao ZM, Tian XS (2012). ABT-737 reverses the acquired radioresistance of breast cancer cells by targeting Bcl-2 and Bcl-xL. J Exp Clin Cancer Res.

[77] Pietenpol JA, Papadopoulos N, Markowitz S, Willson JK, Kinzler KW, Vogelstein B (1994). Paradoxical inhibition of solid tumor cell growth by bcl2. Cancer Res.

[78] Juin P, Geneste O, Gautier F, Depil S, Campone M (2013). Decoding and unlocking the BCL-2 dependency of cancer cells. Nat Rev Cancer.

